# Modeling and Simulation of Either Co-Current or Countercurrent Operated Reverse-Osmosis-Based Air Water Generator

**DOI:** 10.3390/membranes11120913

**Published:** 2021-11-23

**Authors:** Marc Fill, Mirko Kleingries

**Affiliations:** Institute of Mechanical Engineering and Energy Technology, Lucerne University of Applied Sciences and Arts, Technikumstrasse 21, CH-6048 Horw, Switzerland; marc.fill@hslu.ch

**Keywords:** absorption, reverse osmosis, heat transfer, mass transfer, process modeling, air water generation, modeling, simulation

## Abstract

Technologies for obtaining drinkable water are becoming more important as global water consumption steadily increases and climate change progresses. One possibility for obtaining water is the extraction of water vapor from ambient air by means of air water generators (AWG). Previous studies in the field of AWG have mainly dealt with the condensation of humidity on cold surfaces with a cooling system or with absorption and thermal desorption. In this paper, another possibility for AWG is investigated, specifically AWG using absorption and reverse osmosis. For this purpose, models have been set up for an absorber operated in countercurrent and reverse osmosis membrane modules operated in co-current and countercurrent. With these models, simulations with different boundary conditions were then carried out using the programming language Python. The simulations have shown that the reverse osmosis membrane modules operated in countercurrent generally have a lower energy demand and require fewer reverse osmosis stages than those operated in co-current.

## 1. Introduction

Water shortage is a global issue. Competition for fresh water is expected to intensify further as a result of increasing population and climate change [1]. Not only arid regions such as Northern Africa or the Middle East but also, for example, Central and Mediterranean Europe are increasingly affected [2]. According to the World Health Organization and Unicef, half of the world’s population will be living in water-stressed areas by 2025, and only approximately 80% of the world’s population will have access to safe drinking water at home by 2030, leaving 1.6 billion people without [3]. Physically, there is enough fresh liquid water on the earth’s surface in the form of lakes and rivers to supply humankind. This amount corresponds to a volume of about 90,000 km^3^ [4]. However, there are many regions without sufficient natural drinking water resources. In regions where access to seawater or polluted water is available, desalination or wastewater treatment plants can be used to provide clean water [5]. In case seawater is available, reverse osmosis with membranes is normally used due to the high energy efficiency [6]. If no access to liquid water exists, the water must be transported to the recipients by land or air. In many regions affected by water shortages, large facilities that provide clean water in sufficient quantities do not exist, and transport by land or air seems not feasible. Another potential source is the atmosphere, where the water is stored in the form of water vapor. The earth’s atmosphere contains so much water vapor that in its liquid state it would have a volume of about 13,000 km^3^, which is about one seventh the volume of fresh water on the earth’s surface [4].

In order to extract water from the atmosphere, currently either the condensation of air humidity on cold surfaces with a cooling system or sorption with classical desorption (especially by increasing the temperature) is used [7,8]. However, these processes are very energy-intensive. One possible alternative is the combination of two established processes, absorption and reverse osmosis. With a salt solution, water is extracted from the ambient air and subsequently expelled from the aqueous salt solution using multiple reverse osmosis stages instead of desorption. Fill et al. have shown the feasibility of water extraction from the ambient air using this combination [9]. The results in terms of specific energy demand were promising but roughly comparable to those of conventional processes. This makes it necessary to improve the level of detail of the modeling and to investigate process variants. In this way, it can be clarified whether there is potential for savings so that the process can stand out from conventional processes. In the modeling, all state and process variables as well as all key figures are to be recalculated for each finite element. Furthermore, the reverse osmosis process is to be run in countercurrent operation in order to increase the average difference of the chemical potential and thus the water flow through the membranes.

## 2. Materials and Methods

### 2.1. Absorbents

The absorbent utilized in the two sub-processes, absorption and reverse osmosis, has to meet specific requirements. However, these requirements are in direct conflict with one another. On the one hand, the mass fraction of the solute must be maximal to exhibit low water vapor partial pressures in order to absorb enough water vapor from the air, and on the other hand, the mass fraction of the solute must be minimal for low osmotic pressures. For this reason, a variety of different absorbents were evaluated, with aqueous salt solutions containing lithium bromide (LiBr), lithium chloride (LiCl), sodium hydroxide (NaOH), and potassium hydroxide (KOH) proving to be particularly effective. Lithium bromide was eventually chosen for the further investigations because it can attain the lowest water vapor partial pressures and has the lowest toxicity values [9].

### 2.2. Conceptual Design of Reverse Osmosis Based Air Water Generator

**Figure 1:**
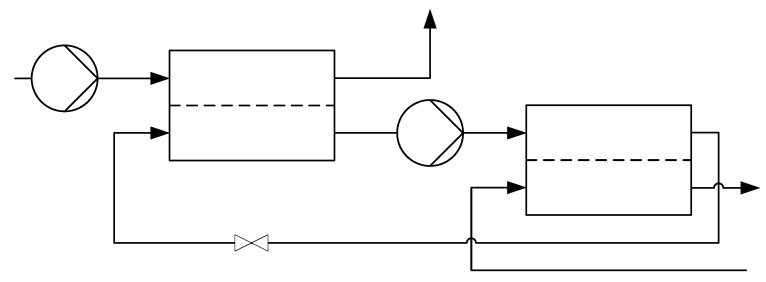
Multi-stage reverse osmosis [9].

Conventional reverse osmosis membrane modules have no inlet on the permeate side and can usually only be operated up to a maximal pressure of about 120 bar [10]. Since osmotic pressures of up to 2000 bar have to be overcome for salt solutions with salt mass fractions of up to w≈0.5, Fill et al. presented a configuration that still allows reverse osmosis to be used [9]. In order to reduce the osmotic pressure, an inlet is added on the permeate side, where a salt solution of lower concentration than in the feed is introduced. Figure 1 shows an exemplary representation of this configuration. The slightly lower concentrated permeate of the first module enters the second module as feed and the retentate of the second module is subsequently reintroduced into the first module as permeate inlet.

This configuration reduces the osmotic pressure across the membrane, which can be calculated from the chemical potential using the following Equation [11] p. 306.

(1) $$\Pi =p_{2}-p_{1}=-\frac{R\;T}{{\tilde{V}}_{j}}\ln\frac{a_{j,2}}{a_{j,1}}=-\frac{R\;T}{{\tilde{V}}_{j}}\ln\frac{x_{j,2}\;\gamma_{j,2}}{x_{j,1}\;\gamma_{j,1}}$$

This modification distributes the osmotic pressure over several modules, but the total pressure to be applied remains the same and thus also the required pump energy. Therefore, pressure exchangers (PX) are used to recover part of the pressure energy. Their efficiency can be calculated as follows.

(2) $$\eta_{PX}=\frac{\sum {\left(p\;\dot{V}\right)}_{out}}{\sum {\left(p\;\dot{V}\right)}_{in}}$$

Pressure exchangers are commonly used in seawater reverse osmosis processes and have an efficiency of up to 95% [12]. By using such pressure exchangers, booster pumps are no longer necessary before each module, employing a booster pump once the pressure falls below a certain limit is sufficient. As a result, there is a massive reduction in the total pressure that must be exerted with pumps.

#### 2.2.1. Co-Current Multi-Stage Reverse Osmosis

**Figure 2:**
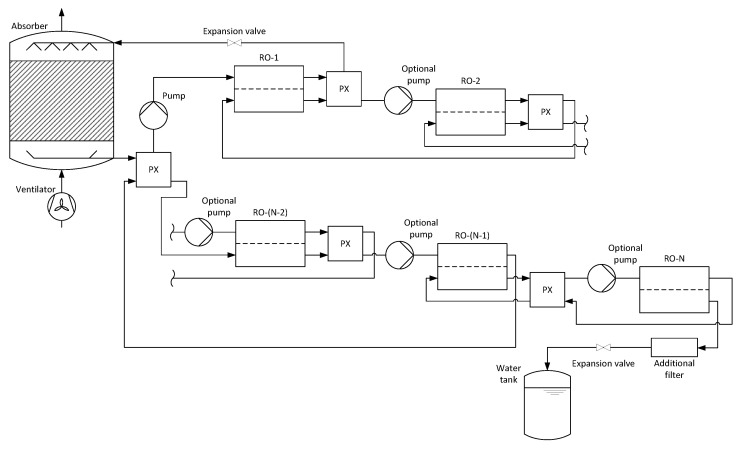
Process schematic for an AWG with absorption and co-current multi-stage reverse osmosis from [9].

Figure 2 shows the schematic of the entire process for an arbitrary number of co-current reverse osmosis membrane modules. A pressure exchanger is placed between each successive pair of reverse osmosis membrane modules. Additionally, one is placed between the second last and first membrane module, which leads to a significant reduction in the overall energy demand of the pumps.

#### 2.2.2. Countercurrent Multi-Stage Reverse Osmosis

**Figure 3:**
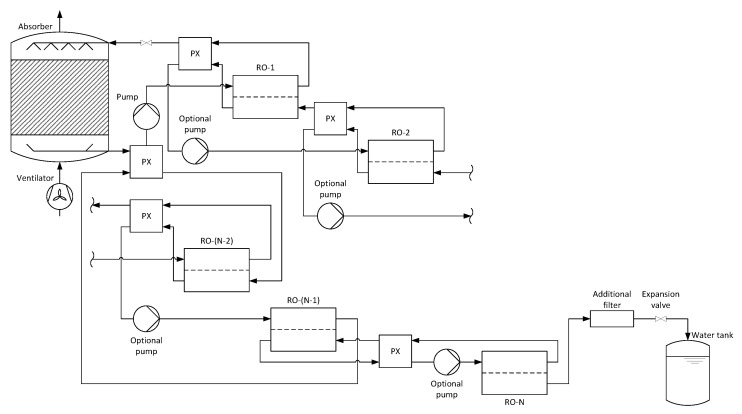
Process schematic for an AWG with absorption and countercurrent multi-stage reverse osmosis.

Figure 3 shows the schematic of the entire process for an arbitrary number of countercurrent reverse osmosis membrane modules. As with the co-current process, a pressure exchanger is placed between two successive and between the second last and first membrane module to reduce the overall energy demand of the pumps.

### 2.3. Modeling

The following models are all assuming steady state operation. The thermodynamic properties of the aqueous lithium bromide solution are determined using Hirschberg’s mathematical fits [13] pp. 788–789. For certain transport properties, mathematical fits of an engineering equation solver from the University of Maryland were used [14]. The vapor pressures of water are calculated using the Antoine Equation [15] p. 439.

#### 2.3.1. Absorber

The absorber is modeled as a falling film absorber, with thin plates arranged side by side providing the surface for the down-flowing salt solution. Air flows into the absorber from the bottom and flows along the salt solution towards the upper end of the absorber; thus the air and the salt solution are in countercurrent flow.

##### Assumptions

To model the absorber, the following simplifying assumptions were made:

- The pressure *p* in the aqueous lithium bromide solution is constant.
- The total pressure $p_{tot}$ of the air is constant.
- The liquid film is flat and has no surface waves.
- The film thickness is considered constant along the height of the absorber column.
- The inlet mass flow rate of the solution and the inlet volume flow rate of the air are assumed to be constant and are calculated according to Appendices B and C of [9].
- The conditions of the air and solution are constant at a given height of the absorber.

These assumptions simplify the model significantly. For a more accurate model, which, however, would also require significant more computational power, the absorber could be discretized in two dimensions or the influence of wave dynamics could be included [16,17].

##### Correlations

In the absorber, a turbulent air flow is desired. According to the VDI Heat Atlas, the Nusselt number for a plane gap with turbulent flow (Re>3×104) can be calculated with the following correlation [18] p. 807.

(3) ζ=1.8log10Re−1.5−2Nu=ζ/8Re−1000Pr1+12.7ζ/8Pr2/3−11+13dhx2/3

The hydraulic diameter $d_{h}$ for a plane gap is defined as $d_{h}=2\;s$, where *s* is the gap thickness. By calculating the Nusselt number at each position *x* in the absorber, position-dependent heat and mass transfer coefficients are obtained.

##### Calculations of the Absorber

The absorber is divided into a finite number of elements *i* and for each of these elements the following calculations are performed.

Before performing the heat and mass transfer calculations, the dry air mass flow rate of the incoming moist air must be calculated, as it is constant throughout the absorber.

(4) $${\dot{m}}_{a,dry}=\left(p_{tot}-p_{w}\right)\frac{{\dot{V}}_{a}}{R_{a}\;T_{a}}=\mathrm{const}.$$

$p_{tot}$ is the total pressure and $p_{w}$ is the water vapor partial pressure of the air.

Similar to the mass flow rate of the dry air, the salt mass flow rate in the absorber remains constant.

(5) $${\dot{m}}_{LiBr}={\dot{m}}_{sol}\;w_{LiBr}=\mathrm{const}.$$

The previously calculated Nusselt number can now be used to calculate the heat transfer coefficient $\alpha_{i}$ and therefore the heat flow ${\dot{Q}}_{i}$.

(6) $${\dot{Q}}_{i}=\alpha_{i}\;A_{i}\;\left(T_{a,i}-T_{sol,i}\right)$$

The water mass flow ${\dot{m}}_{w,i}$ absorbed by the salt solution is calculated with the mass transfer coefficient $\beta_{i}$, which is defined by the dimensionless Lewis number (approximately 1 for an air–water vapor mixture [18] p. 1945).

(7) $${\dot{m}}_{w,i}=\beta_{i}\;A_{i}\;\rho \;\left(X_{a,i}-X_{a,i}^{vap}\right)$$

Therein, $X_{a,i}^{vap}$ is the mass load over the salt solution in kg of water per kg of dry air. With the water mass flow rate ${\dot{m}}_{w,i}$, the enthalpy flow ${\dot{H}}_{i}$ can then be calculated.

(8) $${\dot{H}}_{i}={\dot{m}}_{w,i}\;\left(\Delta h_{v}+\vartheta_{a,i}\;c_{p,vap}\right)$$

With the dry air mass flow rate, heat flow, and enthalpy flow, the energy balance for the air can now be set up and solved for each element *i*.

(9) $${\dot{m}}_{a,dry}\;\left(h_{a,i+1}-h_{a,i}\right)-{\dot{Q}}_{i}-{\dot{H}}_{i}=0$$

Analogously, the energy balance for the salt solution is also set up and solved for each element *i*.

(10) $${\dot{m}}_{sol,i}\;\left(h_{sol,i-1}-h_{sol,i}\right)+{\dot{Q}}_{i}+{\dot{H}}_{i}=0$$

Further, the mass balance for the air is set up and solved for each element *i*.

(11) $${\dot{m}}_{a,dry}\;\left(X_{a,i+1}-X_{a,i}\right)-{\dot{m}}_{w,i}=0$$

Analogously, as with the energy balance, the same is done for the salt solution.

(12) $${\dot{m}}_{LiBr}\;\left(X_{sol,i-1}-X_{sol,i}\right)+{\dot{m}}_{w,i}=0$$

In Equations (11) and (12), $X_{a}$ and $X_{sol}$ denote the mass loads in kg of water per kg of dry air and kg of water per kg of LiBr, respectively.

##### Calculations of the Ventilator

The required electrical power of the ventilator is determined by the pressure losses in the absorber. They can be calculated using the following equation.

(13) $$\Delta p=\zeta \;\frac{l}{d_{h}}\;\frac{\rho \;v^{2}}{2}$$

According to Blasius, the drag coefficient ζ used therein can be calculated for turbulent flows as follows [18] p. 1356.

(14) ζ=0.3164Re4

##### Solution Algorithm

In the solution algorithm, the inlet salt mass fraction and temperature are first assumed. The inlet temperature of the absorber is then adjusted until it matches the outlet temperature and therefore steady state is reached. Subsequently, the inlet salt mass fraction is adjusted until the desired amount of water is absorbed, i.e., the target water output.

#### 2.3.2. Reverse Osmosis Process

The reverse osmosis membrane modules are modeled for co-current and countercurrent flow.

##### Assumptions

For the models of the reverse osmosis membrane modules, the following simplifying assumptions were made:

- No temperature changes over the membranes, the pressure exchangers or the pumps.
- The membrane has a salt rejection of 100%, so no salt flows through the membrane.
- Concentration polarization phenomena in the membrane are not considered.
- Water mass transfer through the membrane is calculated using a membrane constant.
- The representative membrane module used has a pressure drop of 1 bar; therefore, this pressure drop is distributed linearly over the membrane.
- No leakages between the streams in the pressure exchangers.

The membrane of the reverse osmosis membrane module can be modeled with different levels of detail. However, in this work, with the assumptions made previously, the model is simplified. For a more accurate model, which would require more computational power, for example, mass transfer in the membrane could be calculated using the solution–diffusion model, a solute permeability coefficient could be included, or concentration polarization phenomena could be included [19,20,21].

##### Calculations of the Reverse Osmosis Membrane Modules

Before the calculations for the reverse osmosis membrane modules can be performed, the membrane constant $A_{memb}$ must be determined. This can be done using water fluxes specified by the manufacturer under standard conditions [22]. In this model, a representative module from DuPont de Nemours, Inc. was chosen [10].

(15) $$A_{memb}=\frac{J_{w}}{\Delta p-\Pi }$$

Subsequently, the calculations are performed for each element *i*. Since the membrane constant is known, the water flow through the membrane can be calculated:

(16) $${\dot{m}}_{w,i}=A_{memb}\;A_{i}\;\left(\Delta p_{i}-\Pi_{i}\right)$$

where $A_{i}$ is the exchange area, $\Delta p_{i}$ the transmembrane pressure difference between feed and permeate, and $\Pi_{i}$ the osmotic pressure, according to Equation (1), of the *i*-th element. Then the mass flow rate of LiBr in the feed is calculated, which is assumed to be constant since only water passes through the membrane.

(17) $${\dot{m}}_{LiBr,f}={\dot{m}}_{f}\;w_{LiBr,f}=\mathrm{const}.$$

The same applies to the mass flow rate of LiBr in the permeate, which is also constant.

(18) $${\dot{m}}_{LiBr,p}={\dot{m}}_{p}\;w_{LiBr,p}=\mathrm{const}.$$

With the mass flow rate of LiBr in the feed, the mass balance on the feed side can be set up and solved for each element *i*.

(19) $${\dot{m}}_{LiBr,f}\;\left(X_{f,i-1}-X_{f,i}\right)-{\dot{m}}_{w,i}=0$$

Analogously, the mass balance on the permeate side can be set up with the mass flow rate of LiBr in the permeate and solved for each element *i*. For the reverse osmosis membrane module operated in co-current, this results in the following equation.

(20) $${\dot{m}}_{LiBr,p}\;\left(X_{p,i-1}-X_{p,i}\right)+{\dot{m}}_{w,i}=0$$

Similarly, the following mass balance is obtained for the countercurrent reverse osmosis membrane module.

(21) $${\dot{m}}_{LiBr,p}\;\left(X_{p,i+1}-X_{p,i}\right)+{\dot{m}}_{w,i}=0$$

In Equations (19)–(21), *X* denotes the mass load in kg of water per kg of LiBr of the respective membrane side.

##### Calculations of the Pressure Exchangers and Pumps

According to Equation (2), the pressure recovered by the pressure exchanger, respectively, the pressure of the next reverse osmosis module $p_{f}$ can be calculated as follows.

(22) $$p_{f}=\eta_{PX}\;\left(p_{r}+p_{p,out}\right)-p_{p,in}$$

In the previous equation, $\eta_{PX}$ is the efficiency of the pressure exchanger, $p_{r}$ the high pressure retentate leaving the previous module, $p_{p,out}$ the low pressure permeate leaving the previous module and $p_{p,in}$ the entering permeate pressure of the previous module.

To calculate the electric power $P_{el}$ of the pump, the hydraulic power $P_{h}$ and its total efficiency $\eta_{tot}$ are needed.

(23) $$P_{el}=\frac{P_{h}}{\eta_{tot}}=\frac{{\dot{V}}_{sol}\;\Delta p}{\eta_{tot}}$$

The hydraulic power $P_{h}$ of the pump can be calculated from the volume flow rate ${\dot{V}}_{sol}$ of the solution and the required pressure increase Δp [23] p. 48.

##### Solution Algorithm

In the solution algorithm, a salt mass fraction is assumed at the permeate inlet for each reverse osmosis membrane module. Subsequently, this inlet permeate salt mass fraction is adjusted until the osmotic pressure is at a level where the desired amount of water flows through the membrane, i.e., the target water output. A new membrane module is then added, where the inlet salt mass fraction is again adjusted until the desired amount of water flows through the membrane. This is repeated until the osmotic pressure is sufficiently low for pure water to be on the permeate side. Then a conventional reverse osmosis membrane module is added, which has no inlet on the permeate side. Since, due to pressure losses, the available feed pressure at the inlet of a new membrane module steadily decreases, a booster pump is used to increase the pressure as soon as it falls below a certain critical value.

### 2.4. Simulations

The systems modeled according to Section 2.3 were simulated in Python [24]. A water output of 500 L/d, an air volume flow rate of 40,000 m^3^/h, and a solution mass flow of 1.5 kg/s were used as input parameters. Furthermore, different values for the partial pressure of water vapor $p_{w}$ and the ambient air temperature $T_{air}$ were used as boundary conditions for a series of simulations.

For the geometry of the heat and mass transfer area in the absorber, a rectangular cross-section with a height of 3 m, a width of 2 m, and 20 gaps with a gap thickness of 0.05 m were used. For the defined boundary conditions, Equation (13) yields pressure losses Δp of approximately 15 Pa, which corresponds to a required electrical power of 800 W for the specified air volume flow rate [25].

The membrane constant $A_{memb}$ of the used membrane module, which is calculated using Equation (15) and is required for the calculations of the membrane modules, is $3.402\times 10^{-9}$ $\mathrm{kg}/(s\;m^{2}\;\mathrm{Pa})$.

The simulations were carried out for an AWG system with either co-current or countercurrent operated reverse osmosis membrane modules using either a fixed booster pump pressure of p=100 bar or a variable booster pump pressure. With the former, a booster pump is allotted when the pressure drops below 100 bar, and with the latter, the pressure of the booster pump is adjusted to reduce the energy demand per cubic meter of water.

## 3. Results

### 3.1. Co-Current Multi-Stage Reverse Osmosis

**Figure 4:**
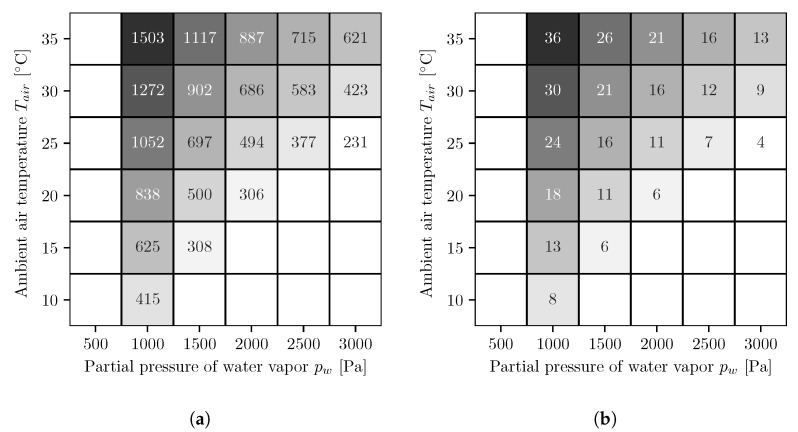

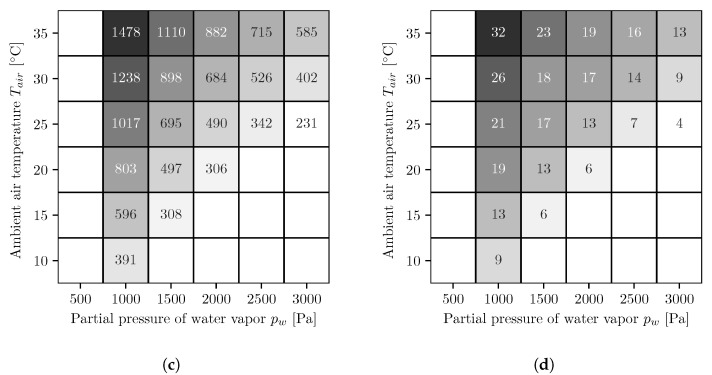
Energy demand per cubic meter of water [kWh/m^3^] (**a**,**c**) and number of necessary reverse osmosis membrane modules (**b**,**d**) for fixed (**a**,**b**) and optimized (**c**,**d**) booster pump pressure. The following applies to all figures. The white areas on the left side represent conditions where, for the chosen absorber dimensions, not enough water can be extracted from the air because the required salt mass fraction is too high and the solution starts to crystallize. To determine whether the solution begins to crystallize, the solid liquid equilibrium of aqueous lithium bromide is used [26]. The white areas in the lower right corners represent conditions where the air is oversaturated and therefore no representative statements can be made.

In Figure 4, the results of the different simulation series for the AWG with co-current operated reverse osmosis membrane modules are shown. The figures on the left side (Figure 4a,c) show the energy demands per cubic meter of water and the figures on the right side (Figure 4b,d) show the number of necessary reverse osmosis membrane modules. The upper ones, Figure 4a,b, are for a fixed booster pump pressure and the lower ones, Figure 4c,d, for variable. At low water vapor partial pressures and high temperatures, the values for the energy demand and the number of modules are both the highest and decrease with increasing water vapor partial pressure and decreasing temperature. The energy demands with the variable booster pump pressure are either equal to or smaller than those with the fixed booster pump pressure.

### 3.2. Countercurrent Multi-Stage Reverse Osmosis

**Figure 5:**
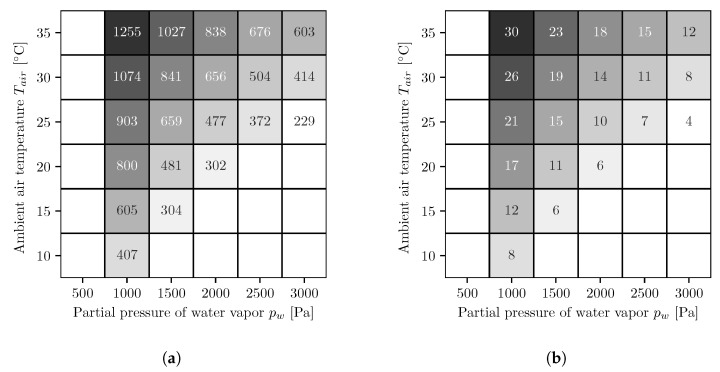

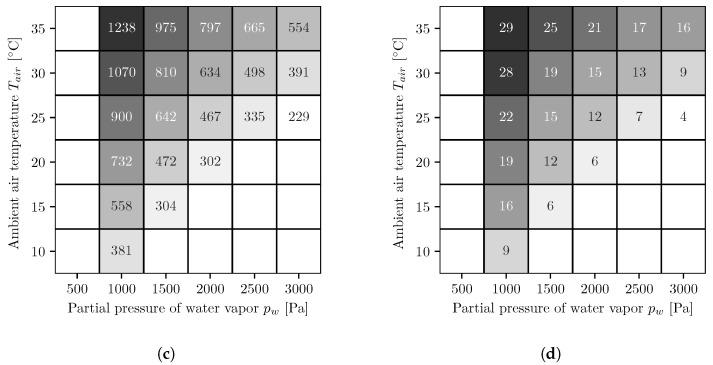
Energy demand per cubic meter of water (kWh/m^3^) (**a**,**c**) and number of necessary reverse osmosis membrane modules (**b**,**d**) for fixed (**a**,**b**) and optimized (**c**,**d**) booster pump pressure. The following applies to all figures. The white areas on the left side represent conditions where, for the chosen absorber dimensions, not enough water can be extracted from the air because the required salt mass fraction is too high and the solution starts to crystallize. To determine whether the solution begins to crystallize, the solid liquid equilibrium of aqueous lithium bromide is used [26]. The white areas in the lower right corners represent conditions where the air is oversaturated and therefore no representative statements can be made.

In Figure 5, the results of the different simulation series for the AWG with countercurrent operated reverse osmosis membrane modules are shown. The figures on the left side (Figure 5a,c) show the energy demands per cubic meter of water, and the figures on the right side (Figure 5b,d) show the number of necessary reverse osmosis membrane modules. The upper ones, Figure 5a,b, are for a fixed booster pump pressure, and the lower ones, Figure 5c,d, for variable. As with co-current, the values for energy demand and number of modules are highest at low water vapor partial pressures and high temperatures and decrease with increasing water vapor partial pressure and decreasing temperature. Likewise, the energy demands with the variable booster pump pressure are either equal to or smaller than the values with the fixed booster pump pressure. Furthermore, the energy demands are smaller compared to the co-current.

## 4. Discussion

In the present paper, AWG systems based on absorption and reverse osmosis were investigated in terms of specific energy demand. The absorber was modeled in countercurrent, whereby the state and process variables as well as the key figures were calculated in each finite element, and the reverse osmosis process was modeled for both co-current and countercurrent operations. Subsequently, these models were combined to systems and used to perform simulations. The simulations were performed for co-current and countercurrent reverse osmosis membrane modules, both with fixed and variable booster pump pressure.

The results of the simulations show similar energy demands for all variants. In the case of co-current reverse osmosis membrane modules and fixed booster pump pressures, the specific energy demand is between ∼230–1500 kWh/m^3^, and for co-current operation with variable booster pump pressures, it is between ∼230–1480 kWh/m^3^. For countercurrent operation with fixed booster pump pressures, the specific energy demand is between ∼230–1260 kWh/m^3^, and for countercurrent operation with variable booster pump pressures, it is between ∼230–1240 kWh/m^3^. The simulations with variable booster pump pressures resulted in energy demands that were equal to or smaller than those with fixed booster pump pressures; however, they required more reverse osmosis membrane module stages. Under ideal conditions, the values simulated in this paper are slightly lower than those determined by Wahlgren for condensation processes, which require between 270 and 550 kWh/m^3^ [27].

Generally, this work has shown that an AWG with absorption and countercurrent reverse osmosis membrane modules is more efficient than one with co-current operation, but under favorable boundary conditions, this has no significant impact on the energy demand. The efficiency of the systems could be further improved, for example, by optimizing the geometries of the components. In the models of the reverse osmosis membrane modules, a salt rejection of 100% was assumed. As this does not apply for real membranes, it should be investigated how strongly salt permeation affects the number of modules and thus the energy demand. In addition, experiments should be carried out to determine whether manufacturing and operating such reverse osmosis membrane modules is possible. Another aspect that has not been considered in this paper is the economic aspect. Therefore, the levelized cost of water (LCOW) should be calculated, i.e., the ratio between the sum of lifetime costs and the sum of lifetime produced water [28]. Next, the LCOW of the process presented in this paper can be compared with other comparable processes and evaluated economically. In addition, further simulations can be carried out with the other previously mentioned absorbents to compare their LCOW with each other.

## Data Availability

Not applicable.

## Abbreviations

AWG	Air water generator/air water generation
LCOW	Levelized cost of water
PX	Pressure exchanger
**Symbols**	
*a*	Activity [−]
*A*	Area $\left[m^{2}\right]$
$A_{memb}$	Membrane constant $\left[\mathrm{kg}/\left(s\;m^{2}\;\mathrm{Pa}\right)\right]$
*c*	Concentration $\left[\mathrm{mol}/m^{3}\right]$
$c_{p}$	Specific heat capacity (at constant pressure) [J/(kgK)]
*d*	Diameter [m]
*D*	Diffusion coefficient $\left[m^{2}/s\right]$
*h*	Specific enthalpy [J/kg]
$\dot{H}$	Enthalpy flow [W]
*J*	Mass flux $\left[\mathrm{kg}/\left(s\;m^{2}\right)\right]$
$L_{char}$	Characteristic length [m]
$\dot{m}$	Mass flow rate [kg/s]
*N*	Number of elements [−]
*p*	Pressure [Pa]
*P*	Power [W]
$\dot{Q}$	Heat flow [W]
*R*	Universal gas constant [J/(molK)]
*s*	Gap thickness [m]
*T*	Temperature [K]
vs.	Velocity [m/s]
*V*	Volume $\left[m^{3}\right]$
$\tilde{V}$	Molar volume $\left[m^{3}/\mathrm{mol}\right]$
$\dot{V}$	Volume flow rate $\left[m^{3}/s\right]$
$w_{i}$	Mass fraction of component *i* [kg/kg]
*x*	Position [m]
$x_{i}$	Mole fraction of component *i* [mol/mol]
$X_{i}$	Mass load of water per component *i* [kg/kg]
**Indices**	
*a*	Air
abs	Absorption
avg	Average
el	Electric
elem	Element
*f*	Feed
*g*	Gas
*h*	Hydraulic
*i*	*i*-th element
in	Inlet
*j*	Solvent
out	Outlet
*p*	Permeate
*r*	Retentate
sol	Solution
tot	Total
vap	Vapor
*w*	Water
**Greek Symbols**	
α	Heat transfer coefficient $\left[W/\left(m^{2}\;K\right)\right]$
β	Mass transfer coefficient [m/s]
γ	Activity coefficient [−]
δ	Film thickness [m]
ζ	Drag coefficient [−]
η	Efficiency [−]
ϑ	Temperature ${[}^{{}^{\circ}}C]$
λ	Thermal conductivity [W/(mK)]
μ	Chemical potential [J/mol]
ν	Kinematic viscosity $\left[m^{2}/s\right]$
ρ	Density $\left[\mathrm{kg}/m^{3}\right]$
Π	Osmotic pressure [Pa]
**Dimensionless Numbers**	
$\mathrm{Le}=\lambda /\left(D\;\rho \;c_{p}\right)$	Lewis number
$\mathrm{Nu}=\alpha \;L_{char}/\lambda$	Nusselt number
$\mathrm{Pr}=\nu \;\rho \;c_{p}/\lambda$	Prandtl number
Re=ρvLchar/η	Reynolds number
